# Estimation of non-constant variance in isothermal titration calorimetry using an ITC measurement model

**DOI:** 10.1371/journal.pone.0244739

**Published:** 2020-12-30

**Authors:** Xiujie Ge, Lan Chen, Dexing Li, Renxiao Liu, Guanglu Ge

**Affiliations:** 1 CAS Key Laboratory of Standardization and Measurement for Nanotechnology, CAS Center for Excellence in Nanoscience, National Center for Nanoscience and Technology, Beijing, P. R. China; 2 University of Chinese Academy of Sciences, Beijing, P. R. China; The University of Alabama, UNITED STATES

## Abstract

Isothermal titration calorimetry (ITC) is the gold standard for accurate measurement of thermodynamic parameters in solution reactions. In the data processing of ITC, the non-constant variance of the heat requires special consideration. The variance function approach has been successfully applied in previous studies, but is found to fail under certain conditions in this work. Here, an explicit ITC measurement model consisting of main thermal effects and error components has been proposed to quantitatively evaluate and predict the non-constant variance of the heat data under various conditions. Monte Carlo simulation shows that the ITC measurement model provides higher accuracy and flexibility than variance function in high *c*-value reactions or with additional error components, for example, originated from the fluctuation of the concentrations or other properties of the solutions. The experimental design of basic error evaluation is optimized accordingly and verified by both Monte Carlo simulation and experiments. An easy-to-run Python source code is provided to illustrate the establishment of the ITC measurement model and the estimation of heat variances. The accurate and reliable non-constant variance of heat is helpful to the application of weighted least squares regression, the proper evaluation or selection of the reaction model.

## Introduction

Isothermal titration calorimetry (ITC) is the gold standard for direct, label-free, and in-situ measurement of complete thermodynamic parameters, including Gibbs free energy **(*ΔG*)**, enthalpy **(*ΔH*)**, entropy **(*ΔS*)**, and heat capacity change **(*ΔC***_***p***_**)** for interactions in solution [1, 2]. In certain cases, it can even be used to obtain the additional kinetic parameters (*k*_*on*_, *k*_*off*_) simultaneously [3–6]. The modern isothermal titration calorimeter designed with power compensation possesses very low detection limits [2, 7] and is widely used in biomolecular interaction studies, supramolecular chemistry, drug research, nanomaterials science, and other fields [8–13]. Thermodynamic information is key in drug design, discovery and optimization [14–16], because it provides details about the balance of driving forces that cannot be obtained solely from current structural and computational methods [17, 18]. Accurate determination of the intrinsic enthalpy and entropy provides the necessary validation data for the development of structure-thermodynamics correlations in rational molecule design [19–22] and extends the understanding of the abstruse enthalpy-entropy compensation [23] and the discrepancies between Van't Hoff and calorimetric enthalpies [24–26].

In a typical ITC titration experiment, the buret with a syringe needle injects the titrant solution into the reaction cell containing the titrand solution at the preset injection volume and time interval to start the reaction. At the same time, the measuring unit detects the thermal power signal of the reaction cell in real time. Each time the titration solution is injected to initiate a reaction, a time-related thermal pulse signal (*dQ/dt*) is generated, and the heat rate curve is integrated over time to obtain the binding isotherm (Q versus molar ratio of titrant to titrand). The optimal experimental design is heavily dependent on the error distribution in the binding isotherm. Over the past few years, continuous efforts in data processing have been devoted to improving the accuracy and reliability of ITC. Wiseman and coworkers [27] defined a dimensionless quantity that governs the shape of the binding isotherm, the Wiseman *c*-value *c = K*_*a*_*C*_*cell*_, where *K*_*a*_ is the association constant of the solution reaction, and *C*_*cell*_ is the concentration of the reactant in the sample cell. They recommended *c*-values ranging from 1–1,000 to ensure the S-shape of the binding isotherm. In cases of very weak binding (*c* → 0) or very tight binding (*c* → *∞*), *K*_*a*_ obtained from direct ITC measurements is not reliable [28, 29]. Biswas and Tsodikov have determined the optimal *c*-value between 5 and 20 since the transition region was well covered by the experimental data in this case [30]. However, this result does not consider the inevitable noise during titration. Broecker and coworkers [31] have quantified the influence of noise by overlaying the specified noise distribution on the simulated ideal binding isotherm, where the isotherm noise conforms to Gaussian distribution with a relative standard deviation of 1% of the maximum heat among injections. The relative standard deviation was assumed to be constant at different *c*-values and an optimal window of 40 < *c* < 100 has been determined based on their model [31]. Hansen and coworkers [32] have calculated an optimal window of 50 < *c* < 500 based on a similar assumption. The non-constant variance of the heat data in actual ITC experiment requires the weighted least squares (WLS) regression [33, 34]. However, the standard protocol for ITC data analysis is nonlinear least squares regression, where the software packages provided by the manufacturers of the instruments assume that the error distribution is constant.

Analysis of variance has also been used to help select and validate the reaction model applied to the heat data, especially when multiple models are available based on the same heat data. A significant challenge in ITC is to select an appropriate mathematical model applied to the heat data, especially when the stoichiometry or binding mechanism are not known beforehand. Herrera and Winnik have respectively used two-site and three-site model to fit the heat data of direct and reverse titrations between DGA (diglycolic acid) and Gd (III) [35]. The fitting curves obtained by both models show good agreement with the heat data, while further residual analysis show that the two-site model causes larger heat residual and stronger oscillating patterns. They used F-tests to distinguish models with different binding stoichiometry and suggested that statistical tests based on variance should be used to evaluate the goodness-of-fit for the reaction models [35]. However, the non-constant variance of heat data potentially limits the evaluation and interpretation of the goodness-of-fit. A more accurate and universal heat variance estimation method will be more conducive to the validation and selection of the reaction model.

The variance function can quantitatively describe the error of heat data through an empirical formula, which generally includes two or three items [33, 34, 36], as shown below:

$$\sigma_{i}^{2}=\sigma_{b}^{2}+\sigma_{p}^{2}Q_{i}^{2}+{\left(\sigma_{v}^{}/V_{i}\right)}^{2}Q_{i}^{2},$$ (1A)

$$\sigma_{i}^{2}=\sigma_{b}^{2}+{\left(\sigma_{p}^{}Q_{i}^{}\right)}^{2},$$ (1B)

$$\sigma_{i}^{2}=\sigma_{b}^{2}V_{i}+\sigma_{p}^{2}Q_{i}^{2}+\sigma_{v}^{2}{\left(Q_{i}/V_{i}\right)}^{2}.$$ (1C)

where *V*_*i*_ and *Q*_*i*_ are the injection volume and reaction heat for the i^th^ injection, respectively. The first term in Eq (1) describes the background noise, the second term describes the proportional error together with the third term, which is related to the injection volume error. The above variance function contains the basic error components associated with the instrument, where *σ*_*b*_ is loosely related to the titration material. Tellinghuisen and coworkers have reported and sifted through several variance functions to describe the non-constant variance by performing a residual-based global analysis of 321 heat data from 35 data sets recorded by the VP-ITC instrument (Malvern Panalytical) [33, 37]. The selected variance function is Eq (1A) with *σ*_*b*_ = 3.22(33) μJ, *σ*_*p*_ = 0.00234(20) and *σ*_*v*_ = 0.0154(30) μL [33]. For the same instrument model, Gilson and coworkers have reformatted the variance function as Eq (1B) with *σ*_*b*_ = 0.54 μJ and *σ*_*p*_ = 0.01 at 27°C [36]. Based on the saturated titration heat of succinic acid solution into excess NaOH solution, the third term associated with the injection volume error has been excluded from their evaluation and analysis [36]. Li and coworkers have reformatted the variance function by both CaCl_2_/EDTA saturated titration and water blank titration recorded by NanoITC Standard Volume (TA Instruments Waters-LLC) [34]. Based on the 9 heat variances, where each one is calculated from 60 injections, the fluctuation magnitude of the background noise was found to be positively correlated with the injection volume and the injection volume error is non-negligible in the variance function [34]. The non-constant variance of heat data can be approximated best by Eq (1C) with *σ*_*b*_ = 0.1771(95) μJ∙μL^-0.5^, *σ*_*p*_ **=** 0.00309(22), and *σ*_*v*_ = 0.0214(21) μL [34]. However, all of the current variance function contains only basic error terms originated from the instrument and cannot account for other relevant error sources, e.g. the concentration uncertainty of the titrant solution. Chodera and coworkers have applied Bayesian statistics to analyze ITC data and found that adopting Bayesian credible intervals can describe the variance between independent experiments more accurately than the confidence intervals by the standard nonlinear least squares fitting due to the inclusion of concentration uncertainty [38]. This finding is consistent with a large-scale survey (ABRF-MIRG’02) in which the variation for the binding constants and enthalpies is more than an order of magnitude larger than that reported by the individual participant [39]. This large variation has mainly been attributed to the error in titrant (syringe reagent) concentration fluctuation and estimated about 10% larger [40]. Such large concentration error is non-negligible. Although more precise concentration is systematically achievable, e.g. 1% [36, 41], such small deviation still has an important effect on the evaluation of heat variance.

In this work, the non-constant variance has been analyzed and predicted by the Monte Carlo sampling method using the ITC measurement model, which incorporates the reaction model for solution reactions and injection model for overflow effect. The approach is consistent with the ISO/IEC Guide 98–3:2008 for evaluation and expression of uncertainty in measurements using a measurement model [42, 43]. The predicted results under various experimental conditions are then compared with predictions from the variance function. The latter was found to fail under certain conditions. The ITC measurement model has also been used to quantitatively analyze the effect of the additional error components originated from the titrant solution fluctuations on the distribution of the heat residual. The experimental evaluation protocol for the basic error components was further optimized. To facilitate the application of this approach, the Python source code for the above model and data processing method are provided in the S1–S3 Files.

## Materials and methods

### ITC measurement model

The thermal effects associated with the titration process in ITC experiments can be divided into three parts, such as reaction heat, dilution heat, and friction heat [5]. According to the previous error analysis results [34], the injection volume error is an important error source, which should be included in the measurement model. The proposed measurement model for the ITC binding isotherm is expressed in Eq (2) and is illustrated in the S3 File.

$$Q=r(V_{inj};C_{syr},C_{cell},V_{cell})+d(V_{inj};C_{syr},C_{cell},V_{cell})+f(V_{inj};R_{inj},T)+Q_{p}+Q_{b}$$ (2)

where the injection volume *V*_*inj*_ is an independent variable, and the total heat *Q* is a dependent variable. The explicit expression of the ITC measurement model demonstrates a strong correlation between the total heat error and the injection volume error. The injection volume *V*_*inj*_ can be expressed as *V*_*inj*_ *= V*_*0*_ *+ V*_*ε*_, where *V*_*0*_ is the injection volume preset by the experimenter, and *V*_*ε*_ is a random variable representing the error component originating from the injection system. Experimental results show that *V*_*ε*_ is independent of *V*_*0*_ and subject to a Gaussian distribution, i.e., *V*_*ε*_ *~ N (0*, *σ*_*v*_^*2*^*)*. *r(V*_*inj*_*;C*_*syr*_,*C*_*cell*_,*V*_*cell*_*)* and *d(V*_*inj*_*;C*_*syr*_,*C*_*cell*_,*V*_*cell*_*)* denote the reaction heat and dilution heat, respectively. *C*_*syr*_ and *C*_*cell*_ denote the reactant concentrations in the syringe and sample cell, respectively. *V*_*cell*_ is the volume of the sample cell. *f(V*_*inj*_*;R*_*inj*_,*T)* is the friction heat, a monotonically increasing function of the injection volume, which depends on many experimental parameters, such as the injection rate *R*_*inj*_, sample temperature *T*, the fluid properties of the solutions, etc. The friction heat term has an unknown complex expression, rendering it difficult to evaluate the individual contributions of various error components. Therefore, the usual treatment method is to subtract the dilution heat and friction heat as the background heat through the blank titration experiment. *Q*_*p*_ is a random variable representing any proportional error other than the first three terms in Eq (2), and can be expressed as *(r(V*_*inj*_*) + d(V*_*inj*_*) + f(V*_*inj*_*))*×*P*, where random variable *P* is independent of *V*_*inj*_ and is generally presumed to follow a Gaussian distribution, i.e., *P ~ N (0*, *σ*_*p*_^*2*^*)*. *Q*_*b*_ is a random variable representing the background noise originating from other uncontrollable and uncharacterized factors, such as stochastic thermal events, ambient temperature changes, electrical and stirring instability, system control algorithms, sampling rates, filter settings, integration algorithm of raw power curve, etc. The results of the blank titration experiment of water titration show that *Q*_*b*_ is positively correlated with *V*_*0*_ and follows a Gaussian distribution, i.e., *Q*_*b*_ ~ *N (0*, *V*_*0*_*σ*_*b*_^*2*^*)* which indicates *V*_*ε*_ contributes minimally to *Q*_*b*_ [34]. It is worth noting that the heat of first injection is significantly less than expected due to the backlash of the stepper motor and the titrant leakage from the syringe during the instrument balancing process [34, 38]. In actual experiments, the first titration data should be excluded from the data analysis.

### Simulation parameters for ITC measurement model under various conditions

To make the comparison between the ITC measurement model and variance function representative, the parameters of the basic error components were set as *σ*_*b*_ = 0.1771 μJ∙μL^-0.5^, *σ*_*p*_ **=** 0.00309, and *σ*_*v*_ = 0.0214 μL [34], which are consistent with the experimental evaluation results for NanoITC Standard Volume (TA Instruments Waters-LLC) instrument and also have reference significance for other ITC instruments. To make the simulation reaction representative, we focus on the classic (1:1) two-component binding reaction. When the titration system is fixed, the specific form of *r(V*_*inj*_*)* can be determined from the injection model and reaction model. The independent binding model for reactions and the instantaneous injection model for the overflow effect of the injection process are demonstrated as specific examples in the S1 and S2 Files. The simulation parameters were fixed so as to best replicate the experimental conditions. The reaction parameters *K*_*a*_ and *ΔH* were 1 × 10^5^ M^-1^ and −40 kJ/mol, respectively. The ratio of titrant concentration in the syringe (*C*_*syr*_) to the titrand concentration in the sample cell (*C*_*cell*_) was 6:1, which facilitates the comparison between different reactions with various *K*_*a*_ or *c*-values. The effective volume of the sample cell was 943 μL, and the volume of the syringe was 250 μL. In order to examine the effects of different experimental conditions on the residual distribution, we varied the values of *K*_*a*_, *C*_*cell*_, *ΔH*, and *V*_*inj*_. The examined *K*_*a*_ values were 1.0 × 10^3^, 1.0 × 10^4^, 1.0 × 10^5^, 1.0 × 10^6^, and 1.0 × 10^7^ M^-1^, *C*_*cell*_ values were 0.5, 1.0, 5.0, 10, and 50 mM, *ΔH* values were −10, −20, −30, −50, and −100 kJ/mol, and *V*_*inj*_ values were 4, 6, 10, and 16 μL. To investigate the effect of any other error that may be introduced during the experimental operation, the error due to concentration or reaction parameters (*K*_*a*_ and *ΔH*) was examined herein. The relative standard deviation of titrant concentration was 1%. The relative standard deviation of *K*_*a*_ and *ΔH* were both 1%. Injection volume (*V*_*inj*_) was fixed as 10 μL, *C*_*cell*_ was fixed at 1 mM (*c* = 100), and the other parameters were the same as the basic simulation parameters.

### Distribution propagation by Monte Carlo method

Usually, the complete analytic formula of *r(V*_*inj*_*;C*_*syr*_,*C*_*cell*_*)* is nonlinear, and the theoretical error analysis is tedious because of the overflow effect. Therefore, intermediate variables were used in the calculations, and the propagation of distribution was implemented by the Monte Carlo method [42, 43], which is widely used by random sampling of the independent variables from their probability distributions. The main steps involved in calculating the variance of heat (*Q*) in the ITC binding isotherm are as follows:

1. Select the standard deviations (*σ*_*b*_, *σ*_*p*_ and *σ*_*v*_) of the basic errors according to the ITC measurement model.
2. Establish a specific expression of the reaction heat according to the material systems being titrated. The thermodynamic parameters (*K*_*a*_, *ΔH* and *n*) and other simulation conditions consistent with the experimental conditions were determined a priori.
3. Sample *Q*_*b*_, *P* and *V*_*inj*_ based on their probability density functions, which are considered as Gaussian distributions with standard deviations (*σ*_*b*_, *σ*_*p*_ and *σ*_*v*_, respectively) mentioned in step 1.
4. Calculate heat *Q* for each injection in the titration experiment using the expressions and parameters from step 2 and the parameters *Q*_*b*_, *P* and *V*_*inj*_ sampled in step 3.
5. Repeat steps 3 and 4 a large number of times (the repetition number was 1 × 10^5^ in this work) to obtain a series of simulated *Q*.
6. Calculate the variance, standard deviation and 95% confidence interval for the simulated *Q*.

The key step for the Monte Carlo method is to establish an ITC measurement model that is in line with the experimental measurements. For the Python implementation of the Monte Carlo method, please refer to the S3 File.

### Optimization of experimental evaluation protocol for basic error components

The basic error parameters (*σ*_*b*_, *σ*_*p*_, *σ*_*v*_) of the ITC measurement model can be estimated in the same way as variance function, which are generally estimated by fitting the residuals of multiple titration curves [33] or using specific experimental design, for example, a saturated titration with uniform heat in the complete titration curve [34, 36]. Here, the latter approach was adopted to eliminate the adverse effects of additional error components, for example, titrant concentration fluctuation or reaction parameter fluctuation, as in our previous work [34]. Since the theoretical value of the reaction heat effect (*Q*) is constant, the mean and variance of the integral heat can be obtained directly without the fitting process of integral heat. High accurate information of reactant concentration and thermodynamic parameters are not required, thus effectively shielding the influence of solution fluctuations on the evaluation of the basic error components. The different heat expectations and variances were obtained by changing the injection volume of the same solution. When the ITC measurement model was known a priori, the previous evaluation protocol [34] could be further optimized to reduce the number of titration experiments. In this work, the optimized protocol consists of only 4 groups of titration experiments, which includes 3 saturated titration experiments with injection volumes of 4, 10, and 16 μL, and a water blank titration experiment with 10 μL injection volume. The sample size (total effective injection number at the specific injection volume) remains at 60 to ensure the accuracy of the variance calculation [44]. The three basic error parameters could be estimated using the WLS method to fit the heat variance data with Eq (1C) [34], which is equivalent to the ITC measurement model for the specific saturated titration experiments.

This optimized error evaluation protocol was validated by the Monte Carlo simulation and experiments. The experimental validation data are from 3 groups of saturated CaCl_2_ /EDTA titration experiments with injection volumes of 4, 10, and 16 μL, and a water blank titration experiment with 10 μL injection volume, as carried out in our previous work [34]. The concentrations of the titrant and titrand solutions were specially designed around 5mM to facilitate the extraction of *σ*_*p*_ and *σ*_*v*_, and the water blank titrations were investigated to improve the measurement accuracy of *σ*_*b*_. The titrations of CaCl_2_ into excess EDTA were performed in 0.1 M MES buffers at pH = 8 to ensure the association constant was large enough. It is worth noting that the titration experiment was generally repeated 1–4 times to reach a sample size of 60. The evaluation results will be compared with the reference [34].

For Monte Carlo simulation, the reaction parameters (*K*_*a*_ and *ΔH*) for the saturated titration were set to 1.0 × 10^9^ M^-1^ and -40 kJ/mol, respectively, close to the real reaction parameters of the validation experiments. The titrant concentration in the syringe and the titrand concentration in the sample cell were both 5 mM. Thus, it can be observed that the latter was in excess. The true values of the error parameters were set as *σ*_*b*_ = 0.1771 μJ∙μL^-0.5^, *σ*_*p*_ = 0.00309, and *σ*_*v*_ = 0.0214 μL. In addition, additional error components were introduced to verify the reliability of the saturated titration method. The relative errors of reaction parameters (*K*_*a*_, *ΔH*) and titrant concentration were both set to 1%. The artificial titration data pool with sample size of 10,000 was generated from the ITC measurement model by the Monte Carlo method. Each sample data consisted of 4 groups of titration experiments mentioned above and was fitted using the WLS method to obtain the values and standard deviations of the three basic error parameters. Each fitting result of the three basic error parameters corresponded to the result of each error evaluation experiment. To investigate whether there is bias in the optimized error evaluation protocol, the mean and standard deviation of 10,000 sets of the fitting results of the three basic error parameters will be compared with the true error values i.e. *σ*_*b*_ = 0.1771 μJ∙μL^-0.5^, *σ*_*p*_ = 0.00309, and *σ*_*v*_ = 0.0214 μL. The reliability of the standard deviation of the three basic error parameters obtained by the WLS method in each error evaluation experiment will be verified further with the Monte Carlo sampling from two aspects. One is to compare the mean of 10,000 sets of the standard deviations obtained by the WLS method in each error evaluation experiment with the standard deviation of the 10,000 sets of the fitting value, because the latter is usually more reliable than the standard deviation estimate using the WLS method, especially in the case of nonlinear fitting. The other is to compare the preset confidence probability of the three confidence intervals of the error parameters with the inclusion frequency at which the 10,000 sets of the three confidence intervals contains the true error values.

## Results and discussion

### Comparison of the standard deviation of heat residual

**Fig 1:**
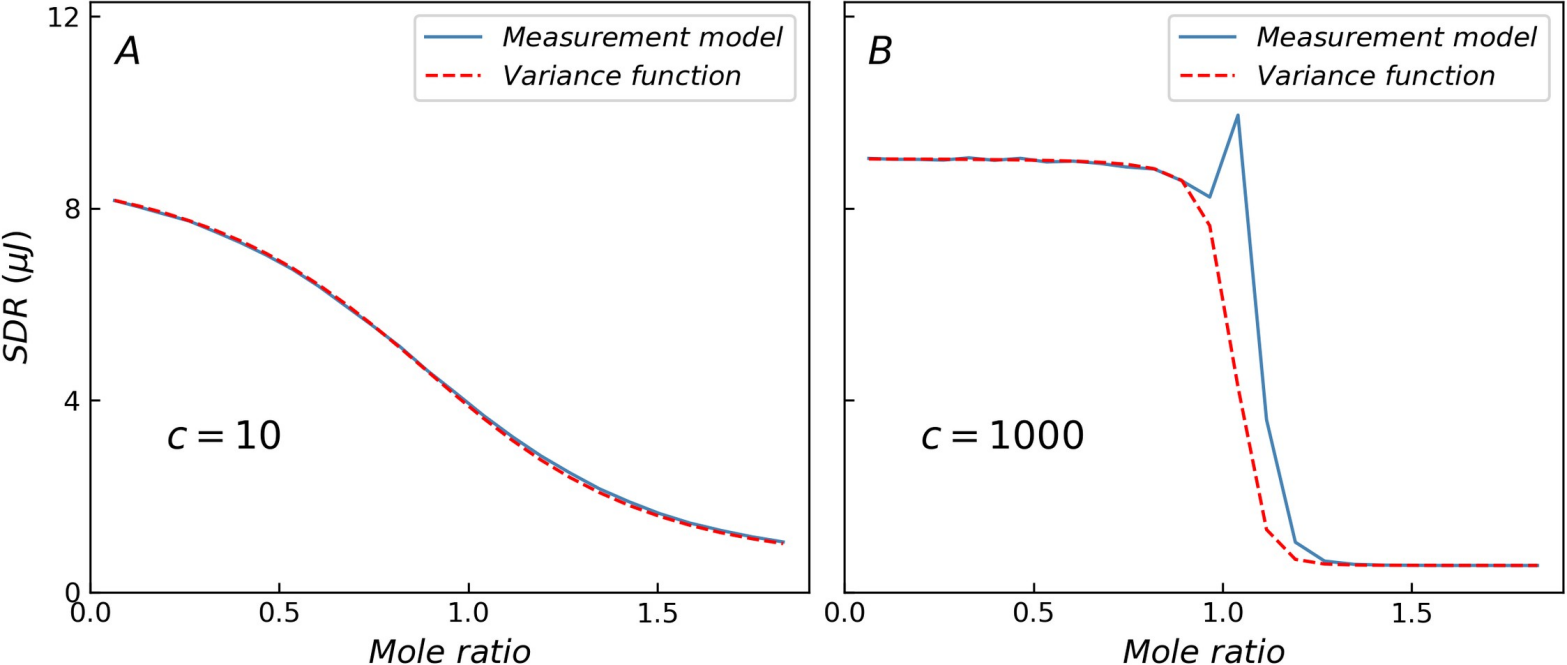
Comparison of SDR predicted by ITC measurement model and variance function. (A) When *c* = 10, the results of two methods are the same. (B) When *c* = 1000, the variance function cannot describe the specific changes in the transition region.

Fig 1 shows a comparison of the standard deviation of heat residual (SDR) obtained from the ITC measurement model (blue solid line) and variance function (red dashed line), where only three basic error components are considered. When *c* = 10, the curves obtained from the two methods nearly coincide. In contrast, when *c*-value is higher, the variance function cannot depict the specific changes in the transition region because of the limitations of the variance function form, which is a monotonically decreasing function and results in lower SDR values in the transition region as shown in Table 1. When *c* = 1000, the residual peak in the transition region (mole ratio around 1) is nearly 3 times the predicted value of the variance function. The non-constant variance may interfere with the statistical tests, especially in the case of the abnormally large residual of high *c*-value reactions in the transition region, which may cause misjudgment in the selection of the reaction model based on analysis of variance. A more realistic and accurate heat variance estimation method will facilitate the selection and validation of the reaction model.

**Table 1:** The maximum deviation of SDR with different *c*-values predict by ITC measurement model and variance function.

*c*-value	10	50	100	500	1000	5000
Maximum deviation (%)	4.0	19.5	36.0	124.5	175.4	261.2

### Non-constant variance predicted by ITC measurement model with only three basic error components

**Fig 2:**
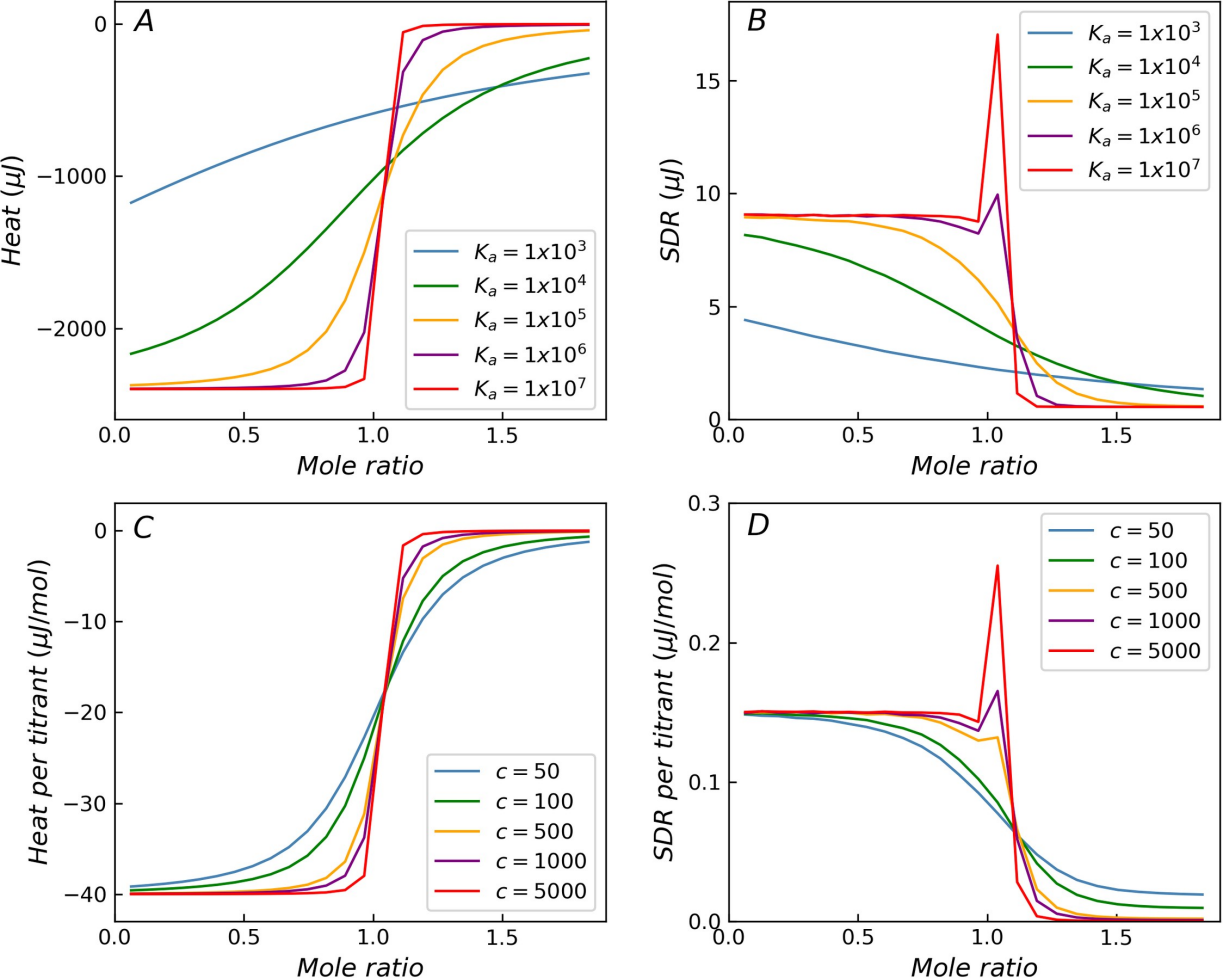
The influence of *K*_*a*_ and *c*-values on the distribution of SDR. Theoretical titration curves and SDR for simulated reactions with different *K*_*a*_ (A, B) and *c*-value (C, D). With the increase of *K*_*a*_ or *c*-value, the SDR increases significantly in the transition region (B, D).

The four primary factors that affect the residual heat, such as *K*_*a*_, *C*_*cell*_, *ΔH* and *V*_*inj*_, were investigated under various conditions for the ITC measurement model using Monte Carlo simulation. Fig 2A shows the theoretical curves of binding isotherms with varying *K*_*a*_ values, when the errors in the experiments are not considered. As the *K*_*a*_ value increases, the transition region of the curve becomes steep. Fig 2B shows the distribution curves of the SDR when only the three error components *σ*_*b*_, *σ*_*p*_ and *σ*_*v*_ are considered. When the value of *K*_*a*_ is low, the distribution of the SDR is monotonically reduced. In contrast, when the value of *K*_*a*_ is higher, the distribution is no longer monotonic, the SDR in the transition region increases significantly, and the distribution on both sides of the transition region remains almost constant. In fact, when the injection volume error is considered, the position of the inflection point in the transition region fluctuates, causing the SDR near the inflection point to vary dramatically. The simulation without injection volume error (*σ*_*v*_) shows that the SDR decreases monotonically in the transition region (As shown in S1 Fig). Therefore, the injection volume error is the reason for the abnormal residuals in the transition region. The ITC measurement model evidently delivers a variance distribution, which is more realistic than that provided by the variance function. In addition, the peak of SDR in the transition region increases and approaches a limiting value with increasing *K*_*a*_. This is consistent with the increasing steepness of the heat distribution curve as it tends toward becoming a step function with increasing *K*_*a*_ (Fig 2A). Fig 2C and 2D shows the simulation results obtained from the ITC measurement model by varying *C*_*cell*_, while keeping the other factors constant (*K*_*a*_ = 1.0 × 10^5^ M^-1^). In addition to the value of *K*_*a*_, the value of *C*_*cell*_ also has a significant impact on the steepness of the binding isotherm, which is ultimately determined by the Wiseman *c*-value (*c* = *K*_*a*_*C*_*cell*_). The significant increase in SDR in the transition region becomes apparent when the *c*-value is higher than 500, and the phenomenon weakens as the *c*-value decreases. The *c*-value is the most important factor to be considered in the heat error distribution in the transition region.

**Fig 3:**
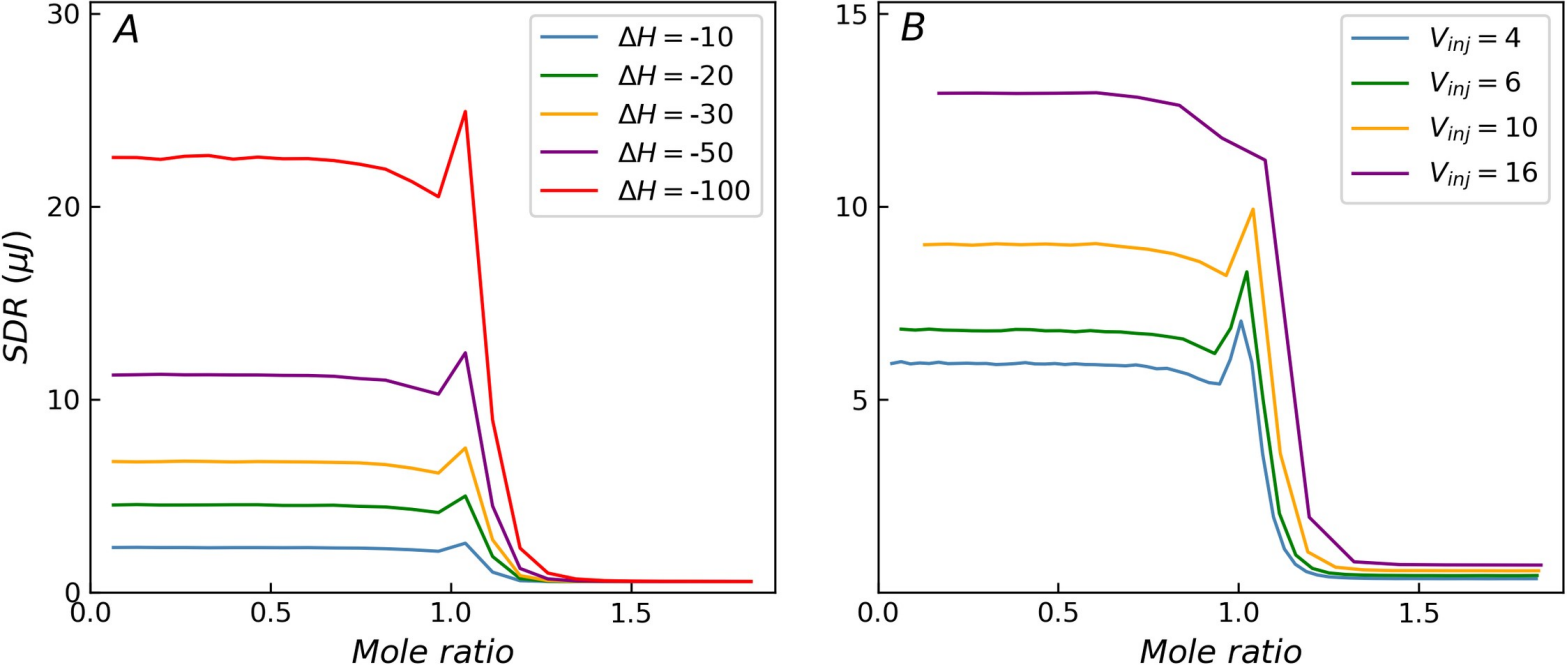
The influence of Δ*H* and *V*_*inj*_ on the distribution of SDR with *c* = 1000. (A) As Δ*H* decreases, the heat residual decreases until the heteroscedasticity disappears. (B) When the injection volume is large, the SDR peak weakens.

When only the *ΔH* of the titration system is varied, the curves corresponding to different *ΔH* values exhibit similar shapes, as shown in Fig 3A. The SDR values reach a peak around the transition region (mole ratio around 1) when the *c*-value is high. As *ΔH* increases, both heat and heat residual increase. The height of the peak in the transition region remains constant relative to the residual at the beginning of the titration curve and continues to increase relative to the residual at the end of the titration curve. When *ΔH* is very low, the background noise term (*σ*_*b*_) dominates, while the other two terms (*σ*_*p*_, *σ*_*v*_) can be ignored. At this time, the heteroscedasticity (non-constant variance) degenerates to homoscedasticity, and the distribution of SDR degenerates to a horizontal line. For homoscedasticity, both ITC measurement model and variance function can deliver a reasonable distribution of SDR. For incremental titration, *V*_*inj*_ value is usually set between 2 and 16 μL. *V*_*inj*_ exhibits some influence on the shape of the SDR distribution curve, but the effect is not as significant as that of the *c-*value, as shown in Fig 3B. When the injection volume is less than 10 μL, though the relative height of the SDR peak varies with respect to the residuals at the beginning of the titration curve, the peak characteristics are preserved in case of higher *c*-values. It is worth noting that when the injection volume is large, the SDR peak in the transition region weakens. For example, when *V*_*inj*_ = 16 μL, the relative error of the injection volume is about 0.1% (*σ*_*v*_/*V*_*inj*_ = 0.0013), and the SDR peak in the transition region almost disappears, shown as purple curves in Fig 3B.

### Influence of solution fluctuations

When the experimental process is disturbed by other factors, additional important errors may be introduced. For example, the solution preparation process is a key step in the titration experiment. When a pipette is used for solution configuration, substantial concentration errors are usually introduced, resulting in significant additional error. Using a balance and density meter instead can effectively reduce the concentration error. In case of biological samples, such as proteins, problems related to activity changes and non-identical batches can easily introduce additional concentration error. In addition, the type, concentration and pH of the buffer may also interfere with the reaction parameters of the material systems, such as *K*_*a*_ and *ΔH*, thereby introducing additional errors into the experiment. The Monte Carlo method based on the ITC measurement model can be seamlessly extended to investigate the influence of these additional experimental errors.

**Fig 4:**
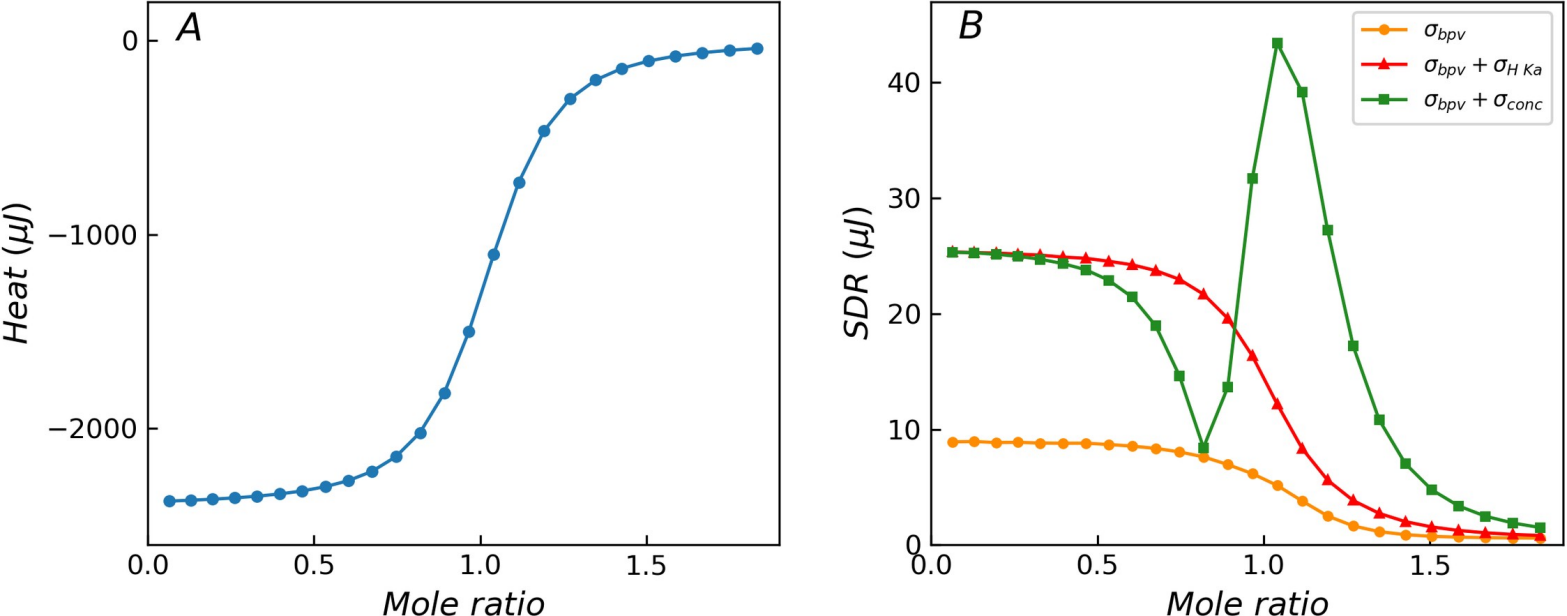
Theoretical titration curves without error components (A) and distribution of SDR for reactions with different error components (B). The orange curve is the control curve with three basic error components (*σ*_*bpv*_), the red curve has additional error components (*σ*_*H Ka*_) in reaction parameters *K*_*a*_ and *ΔH*, and the green curve has additional error component (*σ*_*conc*_) in titrant concentration. The additional error components obviously change the distribution characteristics of the heat residual, resulting in a significant deviation from the prediction result of the variance function.

Fig 4 shows the effect of additional error components of reaction parameters (*K*_*a*_ and *ΔH*) or titrant concentration on the distribution of SDR. The orange curve represents the control curve with only three basic error components (background noise (*σ*_*b*_), heat proportional error (*σ*_*p*_), injection volume error (*σ*_*v*_)). The red curve denotes the additional effects of the reaction parameter error components (1% relative error in *K*_*a*_ and *ΔH*). Major variations are observed between the beginning region of the titration curve and transition region in which the chemical reaction occurs. The additional error components enhance the heteroscedasticity. The green curve shows the additional effects of typical concentration error (1% relative error in titrant) in addition to the control curve. A significant increase can be observed in the SDR values in the beginning of the titration and the wider transition region. A small concentration error (1% in titrant) significantly enhances the heteroscedasticity, and the resulting SDR distribution curve is considerably different from the variance function. This phenomenon will become more prominent when pipettes are used for solution configuration. This implies that the control of the purity and concentration of the reactants is important during the comparison of batch experiments. The ITC measurement model provides a convenient approach to assess the impact of these additional errors on the heat residual, which is difficult to achieve with the variance function.

### Validation of optimized protocol for the basic error estimation

As mentioned above, the additional error components have a significant effect on distribution of heat residual, thereby adversely affecting the evaluation of the basic error parameters. However, an elaborate experimental design and operation may eliminate the interference of these additional errors, as shown in our previous work for the basic error estimation for variance function [34]. The ITC measurement model can be utilized to optimize and validate the experimental protocol further.

**Fig 5:**
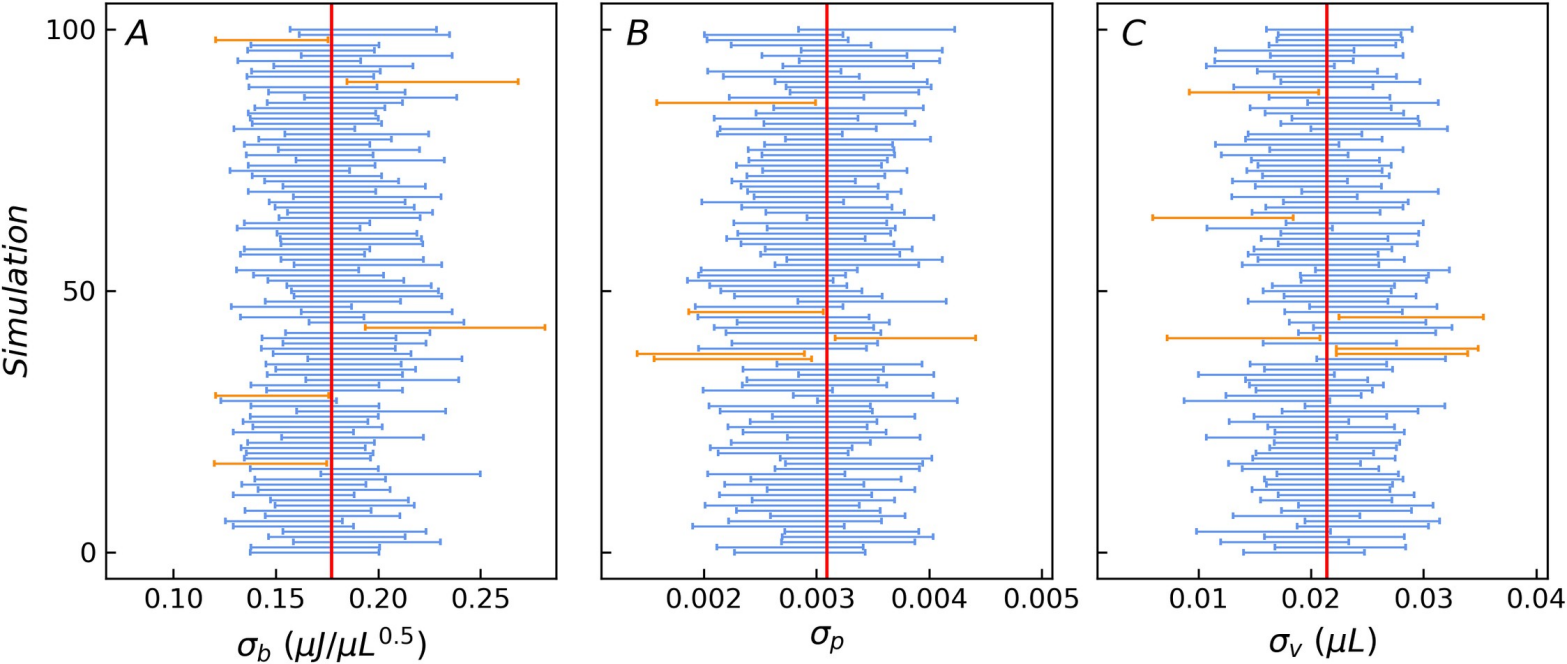
The 95% confidence interval estimates of the three error parameters for 100 samples. The vertical red lines are the true value of the three error parameters. The blue bars indicate the 95% confidence intervals (total four standard deviations wide) containing the true value, while the orange bars indicate the opposite.

The statistical results of the 10,000 sets of the fitting values of the three basic error parameters obtained from the Monte Carlo simulations are *σ*_*b*_ = 0.176(16) μJ∙μL^-0.5^, *σ*_*p*_ = 0.00304(32), *σ*_*v*_ = 0.0212(29) μL, which are consistent with the true values i.e. *σ*_*b*_ = 0.1771 μJ∙μL^-0.5^, *σ*_*p*_ = 0.00309, and *σ*_*v*_ = 0.0214 μL within 2–3 significant digits. The means of the 10,000 sets of the standard deviations obtained by the WLS method are mean(*σ*_*b*__sd) = 0.0164 μJ∙μL^-0.5^, mean(*σ*_*p*__sd) = 0.000319, mean(*σ*_*v*__sd) = 0.00294 μL, which are consistent with the standard deviation of the 10,000 sets of the fitting values std(*σ*_*b*_) = 0.0163 μJ∙μL^-0.5^, std(*σ*_*p*_) = 0.000315, std(*σ*_*v*_) = 0.00294 μL. To compare the confidence probability of the confidence intervals with the inclusion frequency of the true values, Fig 5 illustrates 100 data of the estimated 95% confidence intervals for the three basic error parameters, subsampled from the 10,000 sets. The most intervals include the truth values. Table 2 lists the inclusion frequency of the true error parameters for the confidence intervals estimated by the optimized protocol at three common confidence levels based on the whole 10,000 sets. It is clear from the table that the inclusion frequency is equivalent to the specified confidence levels. In the optimized protocol, only four groups of titration experiments are needed for error parameters evaluation, which greatly reduces the workload and is conducive to the promotion of the method. In addition, it still reduces the potential adverse effects of fluctuations in concentration and reaction parameters.

**Table 2:** The inclusion frequency of true values evaluated by Monte Carlo simulation at three confidence levels for the optimized protocol.

confidence level	inclusion frequency of true values		
	*σ*_*b*_	*σ*_*p*_	*σ*_*v*_
0.683 (1*σ*)	0.685	0.694	0.683
0.954 (2*σ*)	0.948	0.959	0.958
0.997 (3*σ*)	0.993	0.998	0.998

The proposed optimized protocol was validated by experiments further. The validation data are from the CaCl_2_-EDTA saturated titration experiment carried in our previous study [34]. The evaluation result of the corresponding four groups of titration experiments are *σ*_*b*_ = 0.172(16) μJ∙μL^-0.5^, *σ*_*p*_ = 0.00323(32), *σ*_*v*_ = 0.0213(30) μL for CaCl_2_ (6 mM) / EDTA (5.5 mM) system and *σ*_*b*_ = 0.172(16) μJ∙μL^-0.5^, *σ*_*p*_ = 0.00307(32), *σ*_*v*_ = 0.0208(29) μL for CaCl_2_ (4 mM) / EDTA (4 mM) system. Both are consistent with the previous reported values *σ*_*b*_ = 0.1771(95) μJ∙μL^-0.5^, *σ*_*p*_ = 0.00309(22), *σ*_*v*_ = 0.0214(21) μL based on 9 sets of titration experiments [34]. The consistent error evaluation results obtained from saturated titration experiments with different solution concentrations indicate that the concentration error is effectively eliminated.

## Conclusion

A accurate and universal measurement model was established to quantify the non-constant variance of heat data in ITC in this study. Based on the priori three basic errors, the standard deviation of each heat residual in the binding isotherm was well predicted under various conditions by varying *C*_*cell*_, *V*_*inj*_, *ΔH*, or *K*_*a*_. It was revealed that the Wiseman *c*-value and *σ*_*v*_ are the most important factors governing the residual in the transition region. Though the variance function was effective in low *c*-value reactions, it failed to describe the significantly increased variance in the transition region for high *c*-value reactions. In addition, the ITC measurement model also provides a convenient approach to assess the impact of additional errors on the heat residuals. Both the Monte Carlo simulation and titration experiment verified that the optimized protocol can effectively estimate the basic error parameters, eliminate interference of additional error components and reduce the evaluation workload to 4 groups of titration experiments. The quantitative evaluation of error components based on a universal measurement model is expected to contribute significantly to measurement uncertainty evaluation, reaction model validation, experimental optimization and instrument hardware improvement.

## Supporting information

S1 File Instantaneous injection model. (PDF)

S2 File Independent binding model. (PDF)

S3 File ITC measurement model and heat variance estimation. (IPYNB)

S1 Fig The influence of injection volume error on the heat residual distribution with different *K*_*a*_. The left panel shows that when there is an injection volume error (*σ*_*v*_ = 0.0214 μL), the simulated standard deviation of heat residual (SDR) increases abnormally in the transition region; the right panel shows that the simulated SDR decreases monotonically without the injection volume error (*σ*_*v*_ = 0).
(TIF)
