# Supplementary material for: Estimation of non-constant variance in isothermal titration calorimetry using an ITC measurement model
Source: PLoS One. 2020 Dec 30;15(12):e0244739. doi: 10.1371/journal.pone.0244739 (PMC7773272; doi:10.1371/journal.pone.0244739)
Supplement: S1 File — (PDF) [file pone.0244739.s001.pdf]

S1 Instantaneous injection model

The Instantaneous injection model of the overflow effect is suitable for a system employing instantaneous injection of titrant followed by gradual mixing within the reaction cell (effective volume V_c). The concentration of titrant in cell is X_{i-1} prior to the i-th injection. When volume V_i of titrant with concentration of X_s is injected into the cell by the titration syringe, the same volume of solution in cell is expelled into the overflow region at the existing concentration X_{i-1}. After the subsequent mixing, the reactant concentration changes as follows. The concentration of titrant is

$$X_i = X_{i-1} \cdot (1 - \frac{V_i}{V_c}) + X_s \cdot \frac{V_i}{V_c}, \qquad \text{(S1)}$$

where X_i is the concentration of the titrant in cell after the i-th injection, X_{i-1} is the concentration of the titrant in cell before the i-th injection and the initial concentration of the titrant in cell is zero, i.e. $X_0 = 0$. The concentration of titrand is

$$M_i = M_{i-1} \cdot (1 - \frac{V_i}{V_c}), \qquad \text{(S2)}$$

where M_i is the concentration of the titrand in cell after the i-th injection, M_{i-1} is the concentration of the titrand in cell before the i-th injection and M_0 is the initial concentration of the titrand in cell. Eq. (S1) and (S2) are consistent with the algorithm used in the NanoAnalyze ™ data analysis software supplied by TA instruments.
