# Supplementary material for: Estimation of non-constant variance in isothermal titration calorimetry using an ITC measurement model
Source: PLoS One. 2020 Dec 30;15(12):e0244739. doi: 10.1371/journal.pone.0244739 (PMC7773272; doi:10.1371/journal.pone.0244739)
Supplement: S2 File — (PDF) [file pone.0244739.s002.pdf]

S2 Independent binding model

For a reaction with stoichiometry of n, the following equations describe the independent binding model.

$$K_a = \frac{\alpha}{(1-\alpha)\cdot[X]}, \tag{S3}$$

where K_a is the association constant, α is the fraction of sites occupied by ligand X (titrant), $[X]$ is the concentration of free titrant in cell.

$$X_t = [X] + n\cdot\alpha\cdot M_t, \tag{S4}$$

where X_t is concentration of total titrant in cell, M_t is concentration of total titrand in cell, and n is the number of identical sites. Combining Eq. (S3) and (S4) above gives the quadratic equation

$$\alpha^2 - \alpha\cdot(1+\frac{X_t}{n\cdot M_t}+\frac{1}{n\cdot K_a\cdot M_t}) + \frac{X_t}{n\cdot M_t} = 0. \tag{S5}$$

The only root that is physically reasonable is

$$\alpha = \frac{-b-\sqrt{b^2-4\cdot c}}{2}, \tag{S6}$$

where

$$b = -1 - \frac{X_t}{n\cdot M_t} - \frac{1}{n\cdot K_a\cdot M_t}, \tag{S7}$$

$$c = \frac{X_t}{n\cdot M_t}. \tag{S8}$$

Substituting Eq. (S7, S8) into Eq. (S6) obtains the α expression as follows

$$\alpha = \frac{1}{2}\cdot\left[1+\frac{X_t}{n\cdot M_t}+\frac{1}{n\cdot K_a\cdot M_t}-\sqrt{(1+\frac{X_t}{n\cdot M_t}+\frac{1}{n\cdot K_a\cdot M_t})^2-\frac{4\cdot X_t}{n\cdot M_t}}\right]. \tag{S9}$$

The reaction heat Q of solution at fractional saturation α is

$$Q = \Delta H\cdot n\cdot\alpha\cdot M_t\cdot V_c, \tag{S10}$$

where ΔH is the molar heat of binding. The reaction heat can be calculated for any designated values of ΔH, Ka, n by Eq. (S10). However, the parameter of interest for titration experiment is the change in heat from the completion (i-1)-th injection to the completion of i-th injection. The reactant concentrations (M_t, X_t) vary with the titration process. Therefore, it is obviously necessary to correct the M_t, X_t and displacement

volume when the injection is completed. Finally, the reaction heat of *i*-th titration taking loss of solution from overfill into account is

$$Q_i = \Delta H \cdot n \cdot [\alpha_i \cdot M_{t,i} \cdot V_c - \alpha_{i-1} \cdot M_{t,i-1} \cdot (V_c - V_{inj,i})], \tag{S11}$$

where α_i, $M_{t,i}$ are the fraction of sites occupied by titrant and the concentration of total titrand in cell after the *i*-th injection respectively, α_{i-1}, $M_{t,i-1}$ are those before the *i*-th injection respectively, and $V_{inj,i}$ is the volume of *i*-th injection.
