# Supplementary figures and images for: Estimation of non-constant variance in isothermal titration calorimetry using an ITC measurement model
Source: PLoS One. 2020 Dec 30;15(12):e0244739. doi: 10.1371/journal.pone.0244739 (PMC7773272; doi:10.1371/journal.pone.0244739)

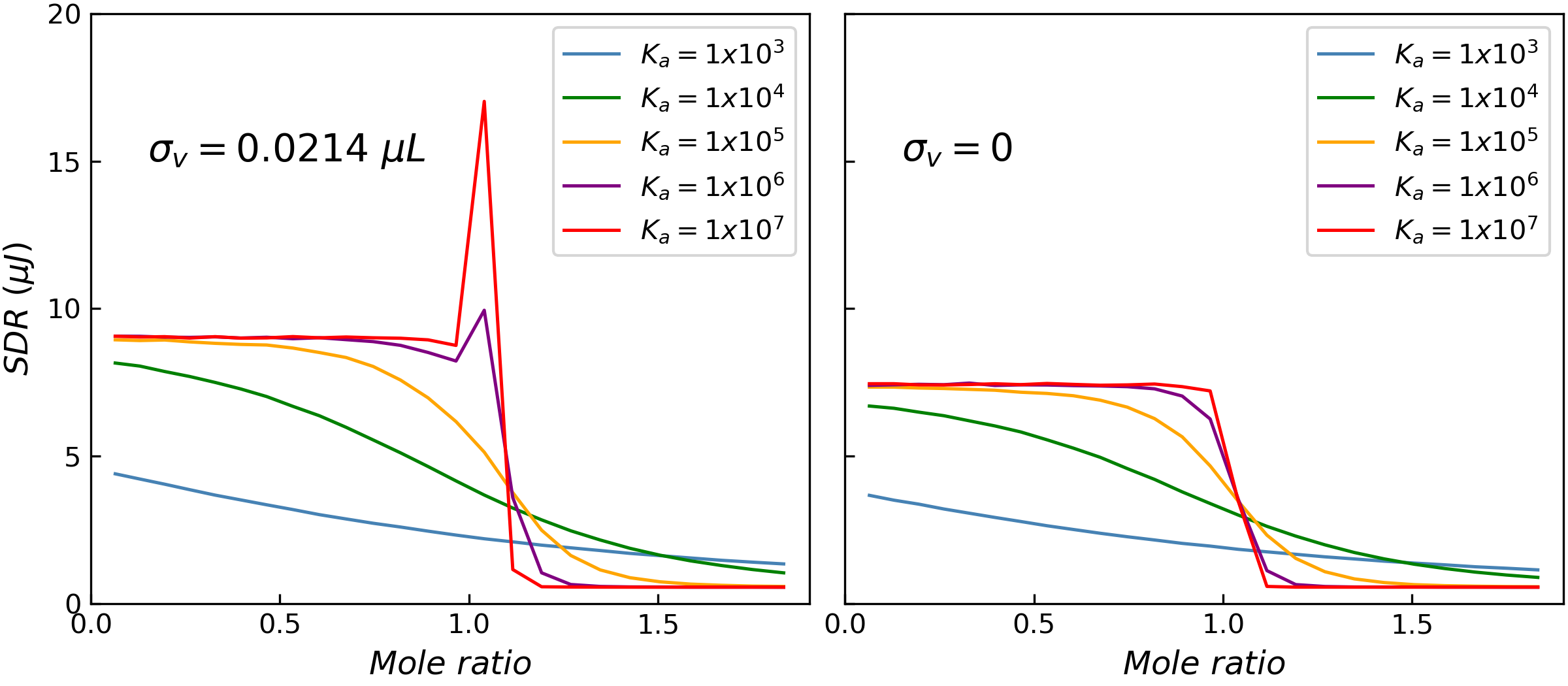

Supplement: S1 Fig — The left panel shows that when there is an injection volume error (σv = 0.0214 μL), the simulated standard deviation of heat residual (SDR) increases abnormally in the transition region; the right panel shows that the simulated SDR decreases monotonically without the injection volume error (σv = 0). (TIF) [file pone.0244739.s004.tif]
